# Predicting the dyadic coping through self-esteem among infertile couples undergoing *in vitro* fertilization and embryo transfer: An actor-partner interdependence model

**DOI:** 10.3389/fpsyg.2023.1127464

**Published:** 2023-08-07

**Authors:** Lihong Zhang, Wei Gu, Xiaoyu Jing, Shihan Zhi, Nan Zhou, Lu Zhang, Wenru Wang, Ying Jiang

**Affiliations:** ^1^School of Nursing, Health Science Center, Xi’an Jiaotong University, Xi’an, Shaanxi, China; ^2^Gynecologic & Reproductive Ward, Northwest Women’s and Children’s Hospital, Xi’an, Shaanxi, China; ^3^Medical College, Xijing University, Xi’an, Shaanxi, China; ^4^Alice Lee Centre for Nursing Studies, Yong Loo Lin School of Medicine, National University of Singapore, Singapore, Singapore

**Keywords:** self-esteem, dyadic coping, infertility, *in vitro* fertilization and embryo transfer, couples, APIM

## Abstract

**Background:**

The population of infertile couples receiving *in vitro* fertilization and embryo transfer in China is increasing gradually. The association of self-esteem and dyadic coping of infertile couples undergoing *in vitro* fertilization and embryo transfer has not been reported. This investigation aimed to examine the predictive effect of self-esteem of infertile couples undergoing *in vitro* fertilization and embryo transfer on coping strategies at the dyadic level.

**Methods:**

A cross-sectional study involving 283 infertile couples was conducted at the Reproductive Center of Northwest Women and Children’s Hospital in China. Participants were asked to complete two self-administered questionnaires, to assess self-esteem (Rosenberg Self-Esteem Scale) and dyadic coping (Dyadic Coping Inventory). Paired *t*-test and Pearson correlation were used to analyze the difference and correlation of variables between wife and husband. The actor-partner interdependence model was used to test the predictive effect of each individual’s self-esteem on their own and their partners’ dyadic coping.

**Results:**

Infertile couples’ self-esteem and dyadic coping are in the medium range. The self-esteem of wives and husbands can fully predict their own dyadic coping. Meanwhile, the husband’s self-esteem can predict the wife’s stress communication (*β* = 0.135, *p* = 0.025), support dyadic coping (*β* = 0.142, *p* = 0.019), and negative dyadic coping (*β* = 0.133, *p* = 0.024), and the wife’s perceived partners’ supportive dyadic coping (*β* = 0.147, *p* = 0.014) and negative dyadic coping (*β* = 0.144, *p* = 0.016). Similarly, the wife’s self-esteem can predict the husband’s supportive dyadic coping (*β* = 0.195, *p* < 0.001), and the husband’s perceived partners’ stress communication (*β* = 0.184, *p* = 0.003) and supportive dyadic coping (*β* = 0.180, *p* = 0.002).

**Conclusion:**

The actor-partner analyses revealed insight into how infertile couples undergoing *in vitro* fertilization and embryo transfer interact and highlighted the importance of self-esteem in dyadic coping styles. Future psychological interventions can enhance self-esteem as an effective way to improve dyadic coping of infertile couples.

## 1. Introduction

Infertility is the inability to achieve a viable pregnancy after 12 months of regular, unprotected sex or as a result of the impaired reproductive capacity of the individual or partner (American Society for Reproductive Medicine, 2020). Worldwide, about 15% of couples of childbearing age have infertility problems (Sun et al., 2019). In China, the proportion has reached as high as 25% and is rising (Zhou et al., 2018). With the advent of human-assisted reproductive technology (ART), a growing number of infertile couples are opting for *in vitro* fertilization and embryo transfer (IVF-ET) to fulfill their fertility wishes (Chen and Heilbronn, 2017). However, high costs, extensive testing and continual monitoring, uncontrollable outcomes, and alternating feelings of hope and disappointment throughout IVF-ET are everyday stressors for both the wife and the husband (Koert and Daniluk, 2017; Casu et al., 2018). These stresses lead to repeated feelings of tension, anxiety, and depression in infertile couples, which seriously affect their quality of life (Fallahzadeh et al., 2019; Dadhwal et al., 2022). Furthermore, chronic stress and adverse mood swings may reduce the chances of a successful outcome through psychobiological mechanisms (Ebbesen et al., 2009; Purewal et al., 2018). Hence, the couple must learn to cope with the range of psychological and physical distress accompanying this transition.

According to Lazarus and Folkman’s (1984) transactional model of stress and coping, coping is a self-regulatory process used to alleviate stress. It consists of intentional cognitive and physical efforts to manage internal or external demands that have been appraised as straining a person’s resources. Studies have shown that women were more likely to adopt emotion-focused strategies than men when coping with the stress of infertility. They reported greater use of self-blame and avoidance, which were associated with higher levels of depression, anxiety, and stress. They also made more effort to seek information and emotional support (Bayley et al., 2009). Partner communication difficulties and active-avoidance coping were predictors of high fertility stress. Men who reported frequently use of active-confronting coping and women who reported moderately or frequently use of meaning-based coping were significantly associated with low fertility stress (Schmidt et al., 2005). Moreover, the coping styles adopted by the partner were related to the personal, marital, and social distress of both men and women (Peterson et al., 2008). In intimate relationships, coping with stress includes individual efforts and mutual cooperation between the two partners.

Bodenmann (1995) further argued that the stress-coping model should not be viewed as an interaction based solely on the individual, but that both partners should perceive stress, evaluate stress, communicate stress, and manage stress together, and therefore proposed the systematic-transaction model (STM). The STM demonstrated that when a couple faces stress from both or one of them, the stress may be transformed into dyadic stress, which then activates dyadic coping due to the interdependence of the two sides. Dyadic coping is a couple’s response and strategy when dealing with dyadic stress. Dyadic coping can be either positive or negative, including stress communication, supportive, delegated, negative, and common dyadic coping (Bodenmann, 1995). Dyadic coping was initially used to highlight the influence of daily stressors on couples and then gradually has been used for cancer patients, chronic disease patients, infertile patients, and their spouses (Vaske et al., 2015; An et al., 2019; Bodenmann et al., 2019; Tang et al., 2022). In the infertile population, infertility and IVF-ET treatment affect both husband and wife as dyadic stressors. Therefore, dyadic coping plays an essential role in determining the outcomes of infertile couples (Casu et al., 2019). Positive dyadic coping strategies can release psychological distress (Patel et al., 2018) and contribute to better marital adaptation and greater relationship satisfaction (Facchin et al., 2021). While there are many benefits to positive dyadic coping, it is important to understand it in infertile people undergoing IVF-ET and find effective ways to improve it. Studies have also found that several factors, including religion, number of miscarriages, family relationships, family intimacy, and adaptability, may influence dyadic coping (Tang et al., 2022).

Self-esteem is the core of self-evaluation, an individual’s positive or negative attitude towards themselves based on self-cognition (Rosenberg, 1965). Self-esteem is an expression of intrinsic value, but it can be influenced by external factors, including physical and psychological conditions (Ucar et al., 2021; Cui C. et al., 2021). Family relationships, psychosocial stress, and physical pain caused by their infertility and treatment are the risk factors that affect the self-esteem of couples undergoing IVF-ET (Zurlo et al., 2020; Jing et al., 2021; Liu et al., 2021; Zhang et al., 2022). Many studies in the past have shown that one of the most prominent problems in the infertile population is stigma, which leads to varying degrees of reduced self-esteem (Cizmeli et al., 2013; Çavdar and Coşkun, 2018; Ruth and Akintayo, 2021; Cui C. et al., 2021). The level of self-esteem has an impact on coping strategies. A high level of self-esteem promotes positive coping behavior. People tend to use problem-focused strategies when they have a higher level of self-esteem. In comparison, they are more likely to use emotional-focused strategies when they have a lower level of self-esteem (Cui S. et al., 2021; Skwirczyńska et al., 2022). That self-esteem, as an expression of an overall sense of self-worth, is related to stress and coping styles (Bodenmann, 1995). Most existing studies have used self-esteem as a mediating variable to explore its protective effect on health outcomes in the context of various stressors from infertility (Kissi et al., 2013; Cui C. et al., 2021). However, little is known about the association between self-esteem and coping strategies among the infertile population undergoing IVF-ET, especially in intimate dyadic relationships. Therefore, we planned to explore the relationship between self-esteem and dyadic coping by providing theoretical evidence for future interventions aimed at improving dyadic coping.

In the dyadic relationship, many variables of the two individuals are interdependent and interact. Actor-partner interdependent model (APIM) is a method to analyze pairwise data (Olsen and Kenny, 2006). The APIM points out that individual dependent variables are affected not only by individual independent variables, that is, actor effects, but also by specific partners’ variables within a certain range, namely partner effects (Kenny et al., 2006; Kenny, 2011). The aim of this study was to investigate the relationship between self-esteem and coping in the dyadic interaction of infertile couples undergoing IVF-ET. Self-esteem was the independent variable and dyadic coping was the dependent variable. We hypothesized that the wife’s and the husband’s self-esteem would predict their own dyadic coping styles and, to some extent, those of their partner’s.

## 2. Materials and methods

### 2.1. Study design and setting

This cross-sectional study was conducted in the Assisted Reproductive Medicine Center Clinic of the Northwest Women’s and Children’s Hospital in Shaanxi Province, China. Infertile couples who were undergoing IVF-ET were recruited between May and July 2022. Data collection took place immediately after obtaining the consent of the participants at the recruitment site.

### 2.2. Participants

Participant recruitment was based on the following criteria. The inclusion criteria were couples who (1) met the WHO definition for infertility diagnosis, that is, one of or both husband and wife are diagnosed as infertile; (2) were receiving IVF-ET treatment; (3) were 20–45 years old; (4) were able to understand and communicate in Chinese. We excluded patients with cognitive and psychiatric illness through interviews. Participants were asked whether they had had ever visited a psychiatric clinic, and if not, included; if so, ask for a specific diagnosis and rule it out if the diagnosis is considered cognitive disorders or psychiatric illness. Diagnostic criteria was based on the Diagnostic and Statistical Manual of Mental Disorders, 5th edition criteria, including anxiety disorders, depressive disorders, eating disorders, obsessive–compulsive disorders, personality disorders, etc.

### 2.3. Sample size

The sample size was estimated using the formula for the cross-sectional study: $n=Z_{\alpha /2}^{2}\sigma^{2}/\delta^{2}$(α = 0.05, $Z_{\alpha /2}^{2}$=1.96; Li and Liu, 2012). Before the formal study, we selected 50 couples for pre-survey, and the average dyadic coping score was 127.958 ± 19.66. With a *σ* of 19.66 and a *δ* of 2% of the average dyadic coping score (127.958), *n* = 227 were obtained by calculation. Considering 20% of invalid samples, 272 dyads couples, consisting of 544 participants, would be adequate. In addition, this study complied with the requirement that the sample size of the structural equation model is at least 200 (Byrne, 2016).

### 2.4. Procedure

The study was approved by the Ethics Review Committee of the Reproductive Medicine Center of Northwest Women and Children’s Hospital (2022007). Participants were recruited in the waiting room of the outpatient department of the Reproductive Medicine Center and were assessed on the day of the IVF-ET appointment. Patients were screened and invited by researchers according to uniform inclusion criteria. All participants were informed of the purpose of the study and the method of participation. If one or both husband and wife refused to participate in the study, the couple was excluded. Only couples who agreed to participate in the study were taken to a separate conversation room. Written informed consent was obtained before data collection. After obtaining informed consent, the researcher delivered the questionnaire to the couples, who completed it independently under the supervision of the researchers. The questionnaire was self-administered and generally took between 20 and 30 min to complete. Sharing and discussion of questionnaire content between husband and wife were not allowed. If there was something unclear, researchers were always available to help and guide. The confidentiality of the data collected and the information of all participants were assured. As a token of appreciation, participants who had completed the questionnaire were asked if they had any needs and we could provide free psychological consultations or IVF-ET treatment-related information consultations if they needed.

### 2.5. Measures

#### 2.5.1. Social demographic characteristics and infertility-related information

The general data collected in this study include age, year of marriage, religion, place of residence, education, occupation and monthly family income. Information related to infertility includes the duration of infertility, whether or not already have a child, the cause of infertility, the length of treatment, and the times of IVF-ET.

#### 2.5.2. Self-esteem scale

Self-esteem is measured by Rosenberg self-esteem scale (RSES) (Rosenberg, 1965; Schmitt and Allik, 2005), which has been widely used in the world. It consists of 10 items ranging from 1 to 4 on a Likert-type scale. The higher the total score, the higher the degree of self-esteem. The total scores below 25 represent low self-esteem, 26–32 represent medium level, and those above 33 represent high self-esteem. The Chinese version of RSES was used in this study, which had a Cronbach’ α between 0.83–0.89 (Chen, 2015). Cronbach’ α in the present study was 0.812 for women and 0.743 for men.

#### 2.5.3. Dyadic coping inventory

The Dyadic Coping Inventory (DCI) (Ledermann et al., 2010) was developed by Bodenmann (2008) based on the systematic-transaction model, which aims to assess dyadic coping and perceived communication of couples under stress. The DCI measures each member’s own dyadic coping behavior and the perceived dyadic coping behavior of the partner through the following five-parts: stress communication, supportive, delegated, negative and common dyadic coping. The DCI is a 37-item instrument and items are rated on a 5-point scale from 1(“very rarely”) to 5(“very often”). The total score of the DCI is the sum from item 1 to item 35 (items 36 and 37 are evaluation items and not included in the total score), in which the negative items are scored in reverse. The scoring standards for DCI are as follows: DCI total score <111, dyadic coping below average; between 111 and 145, dyadic coping in the normal range; >145, dyadic coping above average. In this study, the Chinese version of DCI was used, with Cronbach’ α of 0.51–0.80 of subscales (Xu et al., 2016). Cronbach’α of the DCI in women and men of the present study were 0.920 and 0.939, respectively.

### 2.6. Data analysis

Questionnaires were screened prior to data entry and incomplete and invalid questionnaires (such as all questions that have been selected with the same answer, although some of the questions require reverse answer) would be excluded. The Epidata software (version 3.1; The EpiData Association, Odense, Denmark) was used to input the data and set up the database, the data were analyzed by IBM SPSS software (version 25.0; IBM Corporation, Armonk, NY, United States). Descriptive statistics, such as mean, standard deviation was used to describe continuous variables, while frequency and percentage were used to describe categorical variables. Paired *t*-test was used to analyze the differences between husband and wife in the outcome variables. Bivariate correlation analysis was conducted for all the outcome variables.

In this study, the non-independence of the pairwise data was tested by calculating the correlation of variables between husband and wife. Significant variables in the bivariate correlation analysis were selected as predictive variables to further analyze actor and partner effect in APIM using AMOS software (Version 28.0; IBM Corporation, Meadville, PA, USA). Specifically, taking self-esteem as a predictive variable (W for Wife, H for Husband) and dyadic coping as an outcome variable (W for Wife, H for Husband), the two W variables and the variances of H variables are allowed to be correlated. The paths from W self-esteem to W dyadic coping and H self-esteem to H dyadic coping are called actor effect, and the paths from W self-esteem to H dyadic coping and H self-esteem to W dyadic coping are called partner effect. Gender interaction effects has been investigated before APIM analysis; if there was a significant interaction effect, separate regression analyses were performed for husbands and wives; ultimately, we found no gender differences.

## 3. Results

### 3.1. Demographic data description

We invited 300 couples and 292 of them accepted, with a response rate of 97.3%. After screening, nine pairs of unqualified questionnaires were eliminated, and finally 283 couples consisting of 566 participants were included in the data analysis. The average ages of wives and husbands were 33.43 years old (SD = 4.475) and 32.12 years old (SD = 3.975) respectively. Of these, 67.1% of wives and 73.5% of husbands were between the ages of 30 and 39. More than 50% of couples have been married for less than 5 years. Most of wives and husbands (95%) had no religious beliefs. Nearly 60% of couples lived in cities. Over half of the wives and husbands had college degrees or above. While about 28% of wives were unemployed, only 6% of the husbands were. More than 60% of the couples had a monthly family income between 4,000 and 10,000 RMB (568 to 1,420 USD).

In terms of infertility, more than 86% of couples have been infertile for less than 5 years. The female factor (43.1%) was the most common cause of infertility, followed by unknown factors (26.1%), both factors (16.6%) and male factor (14.1%). About 19% of wives and 15% of husbands already have a child before they were diagnosed with infertility. 74.9% of wives and 65.0% of husbands have undergone different treatments, including medication, surgery, assisted reproduction technology, etc. Approximately 80% of couples were getting IVF-ET treatment for the first time.

### 3.2. Differences in self-esteem and dyadic coping between the husband and wife

The descriptive statistics of the couples’ self-esteem and dyadic coping are presented in Table 1. The means RSES scores reported by both the wives and husbands were within the normal range. Similarly, the means DCI total scores reported by both the wives and husbands were within the normal range.

**Table 1:** Descriptive statistics of self-esteem and dyadic coping scores and results of the paired *t*-test between wives and husbands (*n* = 283 couples).

Variable	Wife mean (SD)	Husband mean (SD)	*r*	*d*	Difference 95% CI		*t*	*P*
					Lower	Upper		
RSES	29.71(3.147)	30.39(3.216)	0.357^**^	0.68(3.607)	0.256	1.101	3.164	**0.002**
DCI	128.18(18.385)	129.01(15.865)	0.589^**^	0.83(15.693)	−1.006	2.667	0.890	0.374
SCO	14.53(2.665)	13.93(2.585)	0.297^**^	−0.60(3.114)	−0.965	−0.236	−3.246	**0.001**
SDCO	18.16(2.864)	18.4(2.623)	0.457^**^	0.24(2.867)	−0.092	0.579	1.431	0.154
DDCO	7.10(1.306)	7.35(1.183)	0.260^**^	0.25(1.517)	0.073	0.428	2.782	**0.006**
NDCO	15.93(2.933)	15.60(3.108)	0.376^**^	−0.33(3.378)	−0.720	0.070	−1.619	0.107
SCP	13.86(2.718)	14.43(2.349)	0.357^**^	0.58(2.889)	0.238	0.914	3.354	**0.001**
SDCP	17.41(3.767)	17.83(3.077)	0.413^**^	0.42(3.753)	−0.019	0.860	1.885	0.060
DDCP	7.29(1.476)	7.26(1.358)	0.272^**^	−0.04(1.713)	−0.236	0.165	−0.347	0.729
NDCP	15.58(3.122)	15.51(2.941)	0.375^**^	−0.07(3.392)	−0.471	0.323	−0.368	0.713
CDC	18.32(3.294)	18.70(2.939)	0.411^**^	0.38(3.396)	−0.023	0.772	1.855	0.065

** At 0.01 level (two-tail), the correlation was significant.

(a) SCO, Stress communicated by oneself.

(b) SDCO, Supportive dyadic coping by oneself.

(c) DDCO, Delegated dyadic coping by oneself.

(d) NDCO, Negative dyadic coping by oneself.

(e) SCP, Stress communication of the partner.

(f) SDCP, Supportive dyadic coping of the partner.

(g) DDCP, Delegated dyadic coping of the partner.

(h) NDCP, Negative dyadic coping by partner.

(i) CDC, Common dyadic coping.The bold values mean that the difference was significant at 0.05 level (two-tail).

### 3.3. Actor-partner interdependence model of self-esteem and dyadic coping

As Table 2 shows, there is a significant correlation in self-esteem and subscales of dyadic coping except for wife-reported self-esteem score and husband-reported score of “negative dyadic coping by partner” (*r* = 0.103, *p* > 0.05), husband-reported self-esteem score and wife-reported score of “delegated dyadic coping by oneself” (*r* = 0.096, *p* > 0.05).

**Table 2:** Analysis of the correlation between subscale scores of dyadic coping and self-esteem.

		Wife’s self-esteem	Husband’s self-esteem
Wife	SCO	0.319^**^	0.228^**^
	SDCO	0.262^**^	0.247^**^
	DDCO	0.151^*^	0.096
	NDCO	0.318^**^	0.225^**^
	SCP	0.208^**^	0.188^**^
	SDCP	0.319^**^	0.243^**^
	DDCP	0.291^**^	0.189^**^
	NDCP	0.287^**^	0.210^**^
	CDC	0.294^**^	0.219^**^
Husband	SCO	0.187^**^	0.285^**^
	SDCO	0.238^**^	0.318^**^
	DDCO	0.189^**^	0.275^**^
	NDCO	0.158^**^	0.331^**^
	SCP	0.194^**^	0.208^**^
	SDCP	0.248^**^	0.300^**^
	DDCP	0.130^*^	0.190^**^
	NDCP	0.103	0.270^**^
	CDC	0.256^**^	0.383^**^

* At 0.05 level (two-tail), the correlation was significant.

** At 0.01 level (two-tail), the correlation was significant.

(a) SCO, Stress communicated by oneself.

(b) SDCO, Supportive dyadic coping by oneself.

(c) DDCO, Delegated dyadic coping by oneself.

(d) NDCO, Negative dyadic coping by oneself.

(e) SCP, Stress communication of the partner.

(f) SDCP, Supportive dyadic coping of the partner.

(g) DDCP, Delegated dyadic coping of the partner.

(h) NDCP, Negative dyadic coping by partner.

(i) CDC, Common dyadic coping.

The bold values mean that the difference was significant at 0.05 level (two-tail).

The actor and partner effects of self-esteem and dyadic coping are shown in Table 3, the results indicated that the self-esteem of wives and husbands has significant actor effects on their own individual dyadic coping (wife-reported: *p* < 0.01, husband-reported: *p* < 0.001). There are partner effects of the husband’s self-esteem on the wife’s individual dyadic coping (*p* < 0.05), expect for “delegated dyadic coping by oneself” (*p* = 0.601). The partner effect of wife’s self-esteem on husband’s individual dyadic coping existed in “supportive dyadic coping by oneself” (*p* < 0.001) and “delegated dyadic coping by oneself” (*p* = 0.05). Similarly, the self-esteem of wives and husbands have significant actor effects on their own perceived partners’ dyadic coping (wife-reported: *p* < 0.001, husband-reported: *p* < 0.01). The husband’s self-esteem has a partner effect on the wife’s perceived “supportive dyadic coping of the partner” (*p* = 0.014) and “negative dyadic coping by partner” (*p* = 0.016). The wife’s self-esteem has a partner effect on the husband’s perceived “stress communication of the partner” (*p* = 0.003) and “supportive dyadic coping of the partner” (*p* = 0.002). Additionally, the wife’s and husband’s self-esteem not only have an actor effect on their own common dyadic coping (wife-reported: *p* < 0.001, husband-reported: *p* < 0.001), but also have a partner effect on their partners’ common dyadic coping (wife-reported: *p* = 0.006, husband-reported: *p* = 0.019).

**Table 3:** APIM analysis predicts wife’s and husband’s individual dyadic coping, perceived partners’ dyadic coping and common dyadic coping from individual self-esteem.

	*B*	*β*	S.E.	*t*	*P*
**Predicting wife’s individual dyadic coping**					
Wife’s self-esteem to Wife’s dyadic coping (Actor effect)					
SCO	0.220	0.260	0.051	4.317	^***^
SDCO	0.227	0.249	0.055	4.142	^***^
DDCO	0.073	0.176	0.026	2.805	0.005
NDCO	0.278	0.298	0.055	5.028	^***^
Husband’s self-esteem to Wife’s dyadic coping (Partner effect)					
SCO	0.112	0.135	0.050	2.244	**0.025**
SDCO	0.126	0.142	0.054	2.354	**0.019**
DDCO	0.013	0.033	0.025	0.523	**0.601**
NDCO	0.122	0.133	0.054	2.249	**0.024**
**Predicting husband’s individual dyadic coping**					
Husband’s self-esteem to Husband’s dyadic coping (Actor effect)					
SCO	0.225	0.281	0.049	4.646	^***^
SDCO	0.239	0.293	0.048	5.024	^***^
DDCO	0.090	0.245	0.022	4.042	^***^
NDCO	0.323	0.334	0.057	5.647	^***^
Wife’s self-esteem to Husband’s dyadic coping (Partner effect)					
SCO	0.070	0.086	0.050	1.420	0.156
SDCO	0.162	0.195	0.049	3.341	^***^
DDCO	0.045	0.119	0.023	1.960	0.050
NDCO	0.079	0.080	0.058	1.345	0.179
**Predicting wife-perceived the partner’s dyadic coping**					
Wife’s self-esteem to Wife-perceived the partners’ dyadic coping (Actor effect)					
SCP	0.184	0.214	0.053	3.467	^***^
SDCP	0.330	0.275	0.071	4.621	^***^
DDCP	0.122	0.261	0.028	4.306	^***^
NDCP	0.245	0.247	0.060	4.097	^***^
Husband’s self-esteem to Wife-perceived the partners’ dyadic coping (Partner effect)					
SCP	0.075	0.088	0.052	1.435	0.151
SDCP	0.172	0.147	0.070	2.461	**0.014**
DDCP	0.049	0.106	0.028	1.753	0.080
NDCP	0.140	0.144	0.058	2.398	**0.016**
**Predicting husband-perceived the partner’s dyadic coping**					
Husband’s self-esteem to Husband-perceived the partners’ dyadic coping (Actor effect)					
SCP	0.119	0.163	0.045	2.672	**0.008**
SDCP	0.242	0.252	0.057	4.242	^***^
DDCP	0.072	0.170	0.026	2.742	**0.006**
NDCP	0.273	0.298	0.055	4.942	^***^
Wife’s self-esteem to Husband-perceived the partners’ dyadic coping (Partner effect)					
SCP	0.137	0.184	0.046	3.006	**0.003**
SDCP	0.176	0.180	0.058	3.029	**0.002**
DDCP	0.048	0.112	0.027	1.807	0.071
NDCP	0.055	0.059	0.056	0.970	0.332
**Predicting common dyadic coping**					
Wife’s self-esteem to Wife’s common dyadic coping (Actor effect)					
CDC	0.284	0.271	0.063	4.538	^***^
Husband’s self-esteem to Wife’s common dyadic coping (Partner effect)					
CDC	0.143	0.140	0.061	2.339	**0.019**
Husband’s self-esteem to Husband’s common dyadic coping (Actor effect)					
CDC	0.321	0.351	0.053	6.107	^***^
Wife’s self-esteem to Husband’s common dyadic coping (Partner effect)					
CDC	0.147	0.157	0.054	2.735	**0.006**

*** At 0.001 level (two-tail), the correlation was significant.

(a) SCO, Stress communicated by oneself.

(b) SDCO, Supportive dyadic coping by oneself.

(c) DDCO, Delegated dyadic coping by oneself.

(d) NDCO, Negative dyadic coping by oneself.

(e) SCP, Stress communication of the partner.

(f) SDCP, Supportive dyadic coping of the partner.

(g) DDCP, Delegated dyadic coping of the partner.

(h) NDCP, Negative dyadic coping by partner.

(i) CDC, Common dyadic coping.

The bold values mean that the correlation was significant at 0.05 level (two-tail).

## 4. Discussion

This study examined the association between self-esteem and dyadic coping among infertile couples undergoing IVF-ET. We adopted the APIM to explore the interdependence of the couples’ self-esteem and their dyadic coping. Overall, our findings supported our hypothesis that the couples’ self-esteem could significantly predict to their own and their partners’ dyadic coping.

Similar to previous surveys in Northeast and Southeast China, we found that the self-esteem of infertile couples undergoing IVF-ET is at the middle level (Cui C. et al., 2021; Wang et al., 2022). In contrast, infertile couples in Turkey (Çavdar and Coşkun, 2018), Athens (Ruth and Akintayo, 2021), and Egypt (Zayed and El-Hadidy, 2020) had low self-esteem and those in western countries such as American, had a higher self-esteem than the general population (Cizmeli et al., 2013). These differences could be the result of socioeconomic and cultural heterogeneity. In general, people of childbearing age in China are more open and receptive to infertility and ART treatment as education, social, economic, and cultural development have improved and knowledge has become more readily available in recent decades. However, there was still a difference in self-esteem between husbands and wives. Previous studies reported that self-esteem is tied to the stress and psychological distress of infertility, and women, as the main body of fertility, face greater reproductive pressure and psychological distress than their husbands, and therefore their self-esteem is more likely to be affected (Cui C. et al., 2021).

The analysis of dyadic coping in our study indicated that wives engaged in more stressful communication than their husbands. Such finding is consistent with earlier studies (Staff et al., 2017; Molgora et al., 2019). As a normal emotional expression, women are more prone to vent their stress to their spouse through words and behaviors in order to receive care and assistance. Conversely, men play a strong and rational role and are typically more emotionally restrained (Bodenmann et al., 2015; Fang et al., 2021). In the current study, husbands reported a better delegated dyadic coping than their wives. The result suggested that although husbands make an effort to assist wives in sharing household duties and to reduce their stress, their efforts may go unrecognized because of the difference between how support is perceived by recipients and how it is perceived by providers (Tang et al., 2022). The better individual dyadic coping in husbands may also reflect men’s socially expected roles as protectors and supporters of their families (Chaves et al., 2021).

The study also found a significant correlation between husbands and wives in self-esteem and dyadic coping, suggesting that individuals’ emotions and behaviors were affected by their partners in the intimate relationship, which supports the findings of Wang et al. (2022). There is a positive predictive effect between self-esteem and dyadic coping, higher self-esteem is associated with more positive dyadic coping of the couple. People with higher self-esteem tend to have better social acceptance, interpersonal relationships and even social resources, so they are more likely to deal with stress positively and provide support to their partner (Alirezaei et al., 2018). On the other hand, research also showed that providing support to the partner could enhance an individual’s sense of self-esteem and well-being, it is one of the effective ways to realize self-worth (Poulin et al., 2010). Getting support is beneficial to enhance self-worth and happiness, as well as adapt to different stressors (Ying et al., 2015). These findings suggested that while self-esteem is associated with a more positive dyadic coping, dyadic coping may also reinforce a person’s self-esteem.

Besides, we found that husband’s self-esteem had a more extensive influence on wife’s dyadic coping. Women are relatively more sensitive and perceptual, which makes their dyadic coping behavior easier to be affected by their husband’s self-esteem (Wang et al., 2022). As the main undertaker of treatment, women bear more psychological pressure and physical pain, and they are eager to get the support and care of their husbands, so they may pay more attention to their husbands’ reactions (Kim et al., 2020). Influenced by Chinese traditional wisdom, women in infertile couples tend to experience more psychological pressure from family and society than men, and are more likely to experience stigma, which persists during fertility treatment (Kong et al., 2019; Zhang et al., 2021). Studies have shown that men are more likely than women to accept childlessness, and the role of motherhood is very important for women, who have a greater sense of responsibility for childbearing (Nagorska et al., 2019). Especially in this study, female factors accounted for a large proportion of infertility, and about 30% of women were unemployed, and the uncertainty of treatment outcomes and financial burden maybe made them feel deeper guilt and sensitivity, and therefore care more about their husbands’ attitudes.

### Limitation

A limitation of the study is related to the design of cross-sectional that could not provide evidence based on causation. However, Crocker et al. (2010) described that cross-sectional research is required when little is known about a phenomenon to guide experimental or prospective research, so it is necessary and interesting to examine the predictive effect of self-esteem on dyadic coping of infertile couples undergoing IVF-ET. An additional limitation is that we focused on the dyadic interaction process of self-esteem and dyadic coping without considering the confounding factors, for example, whether or not already have children, education level, family income etc. Further research can stratify couples undergoing IVF-ET according to different characteristics and explore the dyadic relationship among variables of couples separately. Furthermore, our data collection was conducted on the day of IVF-ET appointment, while studies have confirmed that these variables may change as treatment progresses (Swift and Liu, 2014; Jamil et al., 2020). Hence, longitudinal studies are needed to track the dynamic changes of self-esteem and dyadic coping in the whole IVF-ET process.

### Clinical implication

Findings from the current study highlight the significance of self-esteem as a potential mental resource for infertile couples to manage stress during IVF-ET treatment. Self-esteem may facilitate stress communication and positive dyadic coping in infertile individuals and their partners. Therefore, practitioners working with IVF-ET couples are encouraged to conduct a baseline assessment and help patients in need with psychological counseling. Additionally, the study presented evidence for psychological intervention to infertile couples undergoing IVF-ET that can start with enhancing self-esteem to improving dyadic coping. Based on the fact that infertility and IVF-ET treatment affect couples rather than individuals, as well as the interaction effects between the husband and wife, couple interventions to enhance self-esteem may be effective in improving couples’ ability to cope with stress.

## 5. Conclusion

In summary, this study contributes to the understanding of the relationship of self-esteem and dyadic coping of infertile couples undergoing IVF-ET. Individual’s self-esteem is positively associated with their own dyadic coping and have an impact on their partners’ dyadic coping. Future psychological interventions should focus on enhancing self-esteem to assist infertile couples in positively coping dyadic stress.

## Data availability statement

The raw data supporting the conclusions of this article will be made available by the authors, without undue reservation.

## Ethics statement

The studies involving human participants were reviewed and approved by the Ethics Review Committee of the Reproductive Medicine Center of Northwest Women and Children’s Hospital (2022007). The patients/participants provided their written informed consent to participate in this study.

## Author contributions

LiZ contributed to developing the study design, analyzing data and writing the entire manuscript. WG contributed to developing the study design, reviewing and revising the manuscript. XJ, SZ, NZ, and LuZ contributed to data collection. WW and YJ contributed to revising the manuscript. All authors contributed to the article and approved the submitted version.

## Funding

This study received funding from Shaanxi Provincial Department of Science and Technology (2022SF-438), Teaching Reform Project of Postgraduate Innovation and Entrepreneurship Course of Xi’an Jiaotong University, and Shaanxi Maitian IoT Information Technology Co., Ltd. (SKH2022215). The funders were not involved in the study design, collection, analysis, interpretation of data, the writing of this article or the decision to submit it for publication.

## Conflict of interest

The authors declare that the research was conducted in the absence of any commercial or financial relationships that could be construed as a potential conflict of interest.

## Publisher’s note

All claims expressed in this article are solely those of the authors and do not necessarily represent those of their affiliated organizations, or those of the publisher, the editors and the reviewers. Any product that may be evaluated in this article, or claim that may be made by its manufacturer, is not guaranteed or endorsed by the publisher.
